# Towards an integrated type 1 diabetes management in low-resource settings: barriers faced by patients and their caregivers in healthcare facilities in Ghana

**DOI:** 10.1186/s12913-023-10410-0

**Published:** 2024-01-04

**Authors:** Bernard Afriyie Owusu, David Teye Doku

**Affiliations:** Department of Population and Health, University of Cape Coast, Cape Coast, Ghana

**Keywords:** Non-communicable diseases, Diabetes, Youth, Universal health coverage, Implementation science, Health services research, Electronic health records, iCARE, Integrated healthcare

## Abstract

**Background:**

In Low-Middle-Income Countries (LMICs), young people living with Type 1 Diabetes Mellitus (T1DM) face structural barriers which undermine adequate T1DM management and lead to poor health outcomes. However, research on the barriers faced by young people living with T1DM have mostly focused on patient factors, neglecting concerns regarding plausible barriers that may exist at the point of healthcare service delivery.

**Objective:**

This study sought to explore barriers faced by young people living with T1DM and their caregivers at the point of healthcare service delivery.

**Methods:**

Data were drawn from a qualitative research in southern Ghana. The research was underpinned by a phenomenological study design. Data were collected from 28 young people living with T1DM, 12 caregivers, and six healthcare providers using semi-structured interview guides. The data were collected at home, hospital, and support group centres via face-to-face interviews, telephone interviews, and videoconferencing. Thematic and framework analyses were done using CAQDAS (QSR NVivo 14).

**Results:**

Eight key barriers were identified. These were: shortage of insulin and management logistics; healthcare provider knowledge gaps; lack of T1DM care continuity; poor healthcare provider-caregiver interactions; lack of specialists’ care; sharing of physical space with adult patients; long waiting time; and outdated treatment plans. The multiple barriers identified suggest the need for an integrated model of T1DM to improve its care delivery in low-resource settings. We adapted the Chronic Care Model (CCM) to develop an Integrated Healthcare for T1DM management in low-resource settings.

**Conclusion:**

Young people living with T1DM, and their caregivers encountered multiple healthcare barriers in both in-patient and outpatient healthcare facilities. The results highlight important intervention areas which must be addressed/improved to optimise T1DM care, as well as call for the implementation of a proposed integrated approach to T1DM care in low-resource settings.

**Supplementary Information:**

The online version contains supplementary material available at 10.1186/s12913-023-10410-0.

## Introduction

A few decades ago, diabetes was considered a condition of concern mainly in affluent societies [1]. However, with each passing year, diabetes cases across the globe continue to increase [2]. Current evidence shows that over 537 million people (representing 10.5% of all people across the world) are living with diabetes [2]. In absolute terms, most diabetes cases are in Low-Middle-Income Countries (LMICs) where about 90 per cent of cases are also undiagnosed [2]. The diabetes burden in Africa is already substantial and continues to rise, making it the region with the highest projected increase (134%) in diabetes cases worldwide by 2045 [2]. Ghana is ranked 6^th^ among countries with the highest prevalence of diabetes in Africa [3] and therefore faces no exception to this rapidly increased burden of diabetes, including Type 1 Diabetes Mellitus (T1DM) which is one of the most common endocrine diseases among young people [4].

Despite the increasing global prevalence of T1DM, there is no universal approach to treating patients in healthcare settings [5]. However, there are standards of care which include shortest safe hospital stays, quality assurance standards, self-management education, psychological support, specialised care, multi-disciplinary team of care, provision of essential drugs and logistics, care integration and health promotion [6–8]. Yet, in LMICs such as Ghana, young people living with T1DM face structural barriers including lack of specialist care, insufficient delivery services, erratic supply or excessive costs of life-saving insulin, and other diabetes management logistics such as test strips which remain unobtainable [9–11].

Ghana’s healthcare system is organized into three levels—tertiary (teaching hospitals), secondary (regional and sub-regional hospitals) and primary (health centres and posts). Chronic diseases such as T1DM are clinically managed in tertiary and secondary health facilities due to the availability of improved resources in such facilities [12]. Despite this, there is limited research on barriers to T1DM care in healthcare settings, and related studies have mostly focused on patient factors and coping [9, 11], neglecting healthcare settings and provider-related factors which are equally important. This study therefore sought to explore the barriers faced by young people living with T1DM and their caregivers at the point of healthcare service delivery in Ghana. Exploring and proposing measures to address the health system barriers confronting young patients with T1DM is essential towards achieving Universal Health Coverage (UHC), Health-related Sustainable Development Goals (SDGs), and ultimately improving patients’ health outcomes.

### Theoretical and contextual issues

This research is underpinned by the Chronic Care Model (CCM). One of the most reliable ways of improving healthcare for young people living with chronic diseases is to implement a CCM [7, 8]. This is because models of care tend to emphasize the improvement of healthcare systems through evidence-based research for appropriate healthcare delivery. Thus, the CCM serve as a mechanism through which health systems can be improved to address health concerns. The CCM is an organisational framework created in the mid-1990s by the staff of MacColl Centre for Health Care Innovation, USA, and refined in 1997 by Wagner and his colleagues [13]. It identifies key actors and vital elements needed to provide quality care for patients living with chronic conditions [14–17]. Current evidence indicates that CCM implementation improves health outcomes among diabetes patients [18–20].

The CCM is based on six key components which are: *organization of health care* (i.e. leadership for removing barriers, standards of care, resources)*, self-management support* (i.e. facilitating skill-based education, empowering patients, reminders)*, delivery system design* (i.e. coordinating care processes, defined roles, follow-up)*, decision support* (guiding patients to adopt evidence-based care)*, clinical information system* (i.e. tracking progress, monitoring providers, outcomes and information dissemination to patients)*,* and *community resources* (i.e. primary health care and public health policy) [21]. More prerequisite components are *delivery systems, decision support, self-management support* and *clinical information systems* on which healthcare teams must focus [16, 22]. In this study, we operationalised *community resources* as non-governmental organisations working in the T1DM space since our study focused on healthcare systems.

Recognising the broader policy environment that envelops patients, their families, healthcare organizations, and communities, the WHO recommends an expanded model of the CCM (called the Innovative Care for Chronic Conditions) for healthcare systems worldwide [23]. It is worth noting that research on CCM is mainly from clinical trials and studies conducted in developed countries [24, 25]. This raises concerns regarding its applicability in LMICs largely due to the prevailing healthcare system challenges [26]. Yet, there are opportunities to implement a model of care in LMICs. For instance, in Ghana, *self-management support* for diabetes care via support group services, organizational *reforms* including youth-friendly consultations, *delivery system design* such as periodic hospital reviews, and *clinical information systems* such as diabetes registries are available despite marked constraints including poor coordination, poor records keeping, and weak referral systems. Health system constraints regarding CCM components imply compromised healthcare delivery to patients. Thus, the application of CCM within the context of structural constraints in LMICs may provide meaningful evidence to gauge the capacity of LMICs to implement CCM and identify key change components where T1DM care interventions can yield a cost-effective result.

## Materials and methods

### Study design and selection of study participants

The study was a qualitative investigation into the lived experiences of young people living with T1DM and their caregivers in southern Ghana. Specifically, participants were recruited from regional hospitals and Diabetes Youth Care (DYC) centres in Greater Accra, Central, and Western regions. Major healthcare facilities such as the Korle-Bu Teaching Hospital, the Greater Accra Regional Hospital, the Cape Coast Teaching Hospital, and the Effia-Nkwanta Regional Hospital which are also major referral points for chronic disease management are found in the study area [12]. Descriptive and interpretative phenomenology approaches were used in this study. Twenty-eight young people living with T1DM who self-managed their condition, 12 primary caregivers (PCGs: parents/guardians), six (6) healthcare providers (HCPs: physicians, nurses, pharmacists, diabetes unit heads), and Access to Insulin Care Manager provided data for the study. The young people were located at three source points: support group centres (*n* = 13), their homes (*n* = 9) and hospital centres (*n* = 6).

Participants were recruited into the study using maximum variation, snowball, and convenience sampling techniques. These techniques were informed by the research approach (qualitative) and the phenomenological study design. Concerning the maximum variation technique, participants were selected based on a variety of sociodemographic factors including age, sex, place of residence and support group membership status. Also, through snowball approaches, participants who rarely attended healthcare or support group meetings were identified and interviewed. We used information power (i.e., when sufficient information was collected to address the research questions) to determine the point of saturation. The study employed the inclusion criteria below:

- a. The T1DM patient must be a young person aged within 14 and 24 years residing and receiving T1DM care in the study area. Younger persons were considered to lack the capacity to provide in-depth information about their lived experiences.
- b. Healthcare providers and parents/guardians must have provided/provide continuous T1DM care/support in the study area over the past 24 months preceding the interview. The 24 months continuous were deemed sufficient time to greatly experience the daily life of patients, reduce recall bias, and provide recent and thick descriptions of experiences.

### Data collection instruments and procedure

Three semi-structured interview guides were developed. Each of the interview guide was used to interview young persons living with T1DM, caregivers and healthcare providers respectively. The guides were developed based on the study aims, gaps in literature, and the lead author’s affiliation experience with the DYC. These guides were pre-tested in the study area among 2 young persons living with T1DM, a parent, and a healthcare provider before they were deployed. The main aim of the pre-test was to evaluate participants understanding of the questions. The yield of the pre-test was a refinement of the guides and probing questions. Data for this study were extracted from selected questions from Sections A, B, C and E of the young person’s interview guide, Sections A and E of the caregivers' interview guide, and Sections A, B, C, and E of the healthcare providers interview guide (see Supplementary File: Interview Guide).

Before data collection, the researchers and a DYC coordinator negotiated access to the network of young people living with T1DM through the DYC where the lead author is affiliated as a Volunteer. The lead author introduced the research assistants to the study participants during their monthly psychosocial support group meetings before data collection. This served to facilitate access to the target group and to encourage them to feel comfortable, trust, and share their deep-seated concerns with the research assistants. In the healthcare settings, the interviews were conducted in small conference rooms, as well as rooms used by the DYC for their monthly psychosocial support group meetings.

The study used novel approaches to investigate the subject matter amid COVID-19. Both conventional (face-to-face interviews with 29 participants, observation) and digital data collection methods were used (telephone interviews with 10 participants, videoconferencing with 2 participants, and returned emails with 6 participants). These inclusive methodologies were tailored to the unique setting of participants while adhering to ethical principles of conducting interviews during the global outbreak of COVID-19. For instance, all the healthcare providers (HCPs) completed the interview guide and returned it via email, and a video conference interview (via Zoom) was conducted with an Access to Insulin Care Manager (ACM) for an insulin distribution company in Ghana. In terms of workload management, an interview was conducted by each research assistant per day. The interviews were conducted in English, Fanti, or Twi, depending on which languages participants spoke and understood best. The audio/voice-recorded interviews lasted between 40 and 120 min and were transcribed verbatim into English daily and password protected. Participant's information was coded using an in-vivo pseudonym known as warrior, which is used to describe their struggle to overcome the brunt of T1DM.

### Rigour

Quality assurance mechanisms were embedded during the planning, data collection, and analysis phases. Methods for achieving credibility included observation, extended engagement with participants, triangulation of data sources, participants re-checking through returned transcripts, and independent coding. Confirmability approaches included an investigation of the same issues using a chain of evidence. Others included appropriate research design and methods used in related studies. The use of lengthy descriptions and the presentation of discrepant information allows readers to vicariously reflect on the data themselves. The semi-structured interview guides were guided by a systematic review of the literature, and the lead researchers' regular involvement with T1DM patients and carers. In contrast to closed-ended questions, the open-ended questions encouraged participants to express themselves to encompass the breadth of the issues covered. Also, hospital-setting interviews allowed us to observe some barriers including waiting time, and T1DM care space. In reporting the results, we followed the consolidated criteria for reporting qualitative research (COREQ) [27].

### Data analyses and presentation

Concurrent data collection and analyses were carried out to further aid in determining data saturation. We performed multi-method analyses (thematic and framework analyses) aided by a computer-assisted tool (QSR NVivo 14). The thematic analysis plan followed data familiarisation, inductive coding, identification of themes, and theme comparison. Two research assistants and the lead author coded portions of the transcripts independently and identified themes together with the second author. Further thematic analysis entailed the comparison of different codes/nodes/themes to identify hidden patterns, relationships, and classifications in the data. For example, we compared participants' age, sex, and place of residence with their point of healthcare challenges. We adopted Ritchie et al., (1994) approach to conducting a framework analysis which entailed data familiarisation, identification of a framework, indexing, charting and interpretation [28]. As we wanted our framework to be open to issues that can be applicable, and emerging from the data, we deductively identified the CCM as an important model to theoretically contextualise the qualitative data. We therefore synthesised the themes with the components of the CCM using matrix coding as depicted in Table 2.

### Ethical considerations

The University of Cape Coast Institutional Review Board granted ethical clearance to conduct this study [Clearance ID: UCCIRB/CHLS/2021/19]. Informed consent was obtained from all participants and/or their legal guardians before data collection. During monthly support group meetings, informed consent forms and information sheets were given to the patients who were below age 18 years to be given to their parents/guardians at home to seek their parental consent, and the Coordinators of the DYC followed up with a telephone call to remind parents about the study. Parents who accompanied their children below the age of 18 years to monthly support meetings provided on-site consent for their children. The assent of participants below age 18 years was sought before they were interviewed. All participants above age 18 years provided either written or verbal consent (remote participants) to participate in the study. The study adhered to key COVID-19 protocols during the data collection exercise.

## Results

### Background characteristics of participants

Forty-seven participant representing 28 young people living with T1DM, 12 carers who were parents/guardians, six healthcare providers made up of physicians (*n* = 2), nurses (*n* = 2) and pharmacists (*n* = 2), and an access to insulin care manager were interviewed. There was an equal representation of male and female patients. Their mean age was 20 years, with the lowest and highest ages being 14 and 24 years respectively. The patients had been living with T1DM for about eight years. Fifteen of them had an immediate family history of diabetes (undifferentiated). In terms of marital status, one participant was married, and two others were cohabiting. They commonly received healthcare from regional hospitals, particularly Accra Regional Hospital, Cape Coast Teaching Hospital, Korle-Bu Teaching Hospital, and Effia-Nkwanta Regional Hospital. All the warriors were actively covered under the National Health Insurance Scheme (NHIS). Three participants had newly joined the DYC, and seven of them were irregular meeting attendees.

The warriors mostly sought healthcare services with their carers who were mostly their mothers. The average age of the carers was 45 years, and one of them had a tertiary education. Among the carers, nine were biological mothers of a T1DM patient. Some of the carers also reported a family history of diabetes including T1DM, and four of them were living with diabetes (undifferentiated). Concerning their marital status and economic activity, eight of the carers were married and engaged in petty trading (*n* = 7) respectively. The average duration for T1DM care among the carers was 6 years. Concerning healthcare providers, there were two physicians, nurses, and pharmacists, and they had been directly engaged with TIDM care in their various healthcare facilities for the past 7 years. Detailed socio-demographic characteristics of participants can be found in a related study [7].

### Themes

The inductive analysis yielded eight themes. These themes were: shortage of insulin and management logistics, healthcare providers' knowledge gaps, lack of T1DM care continuity, lack of specialist care, poor healthcare provider-caregiver interactions, shared physical space for diabetes care, long waiting time, and outdated treatment plans. Table 1 summarises these themes, sources, quotes, and participant identities. Quotes are characterized by participant status, age, sex, and years of living with T1DM.

**Table 1:** Thematic framework

Theme	Source	Narratives	Participant
Shortage of insulin and management logistics	19	*There are usual times when the hospital and the pharmacy don’t have insulin*	*Female, 24 years, 13 years of LE*
Healthcare providers' knowledge gaps	7	*Some of them [HCPs] have less than 50% knowledge of diabetes as a “subject”. I remember a nurse saying that I got my diabetes (T1DM) from eating too much sugar and fatty foods*	*Female, 18 years, 17 years of LE*
Lack of T1DM care continuity	6	*He [the doctor] took good care of me until he was transferred. Since then, I haven’t been regular at the hospital because I don’t feel happy going there*	*Male, 24 years, 10 years of LE*
Lack of specialists’ care	6	*There is so much reliance on us [medical doctors] and so we have lost out on training other healthcare providers who should be able to support these young ones and only refer complicated cases to us*	*Physician specialist and DYC Founder*
Poor healthcare provider-caregiver interactions	6	*I told the doctor that all my money is finished, and the doctor, with all his grammar insulted me. He said if I know I can’t take care of the child, why did I give birth to the child?*	*Guardian, 53 years, 3 years of LE*
Shared physical space for diabetes care	4	*When I go to the hospital, I am mixed with the elderly, like around the 40- to 60-year-olds and they scare me. They will be talking amongst themselves saying “eei, this small boy has gotten diabetes, soon they [health care professionals] will cut his leg”*	*Male, 21 years, 10 years of LE*
Long waiting time	4	*I come in the morning at 8:00am, and leave in the afternoon around 3-4 pm*	*Female, 24 years, 9 years of LE*
Outdated treatment plan	1	*Their [hospital] treatment plans are outdated so I rely on recommendations from friends in the USA, France, or Italy*	*Mother, 37 years, 5 years of LE*

*LE* Lived experience

### Shortage of life-saving insulin and test strips

Young people, particularly those who had lived with T1DM for a longer period experienced a lack of T1D management logistics in the hospital settings. They reported that life-saving insulin and other management logistics such as self-monitoring equipment were in short supply at healthcare facilities. These excerpts from participants represent what was said:

*At times, there is a shortage of insulin at the hospital making it difficult to get insulin* [a 21-year-old male warrior with 7 years of lived experience]*.*

While some individuals with T1DM received free glucometers from DYC, its associated test strips were unavailable/difficult to obtain on the market. In explaining this issue, this is what was said:

*Our leaders [DYC] give us machines (glucometers) when we don’t have them. But the problem is that, with the machine they give us, we don’t get the test strips to buy* [a 24-year-old male warrior with 10 years of lived experience]

### Healthcare worker knowledge gaps

Expert patients revealed some knowledge gaps by healthcare providers. This theme was composed of six sub-themes which were: providing care for patients who presented with other signs such as pregnancy, causes of T1DM, fear of insulin prescriptions, fixation on T1DM management, concerns about healthcare worker experimentation, and diet restrictions. Concerning pregnancy, this was what a 24-year-old female warrior with 9 years of lived experience had to say:

*Last two years, I got pregnant and gave birth to a baby boy but because of diabetes, the baby had no ears, his spinal cord was not normal, and he died later. I was not having any support and I went through a bitter experience. I went to* [mentions facility name] *and they told me that the doctors here [hospital where she sought healthcare] did not do a good job because when they identified my condition, they should have performed CS* [Caesarian section] *but it was late. I do not want to go through such situation again.*

It was reported that some healthcare professionals failed to prescribe insulin due to the fear of causing harm to patients. In explaining this issue, this was what the access to insulin care manager had to say:

*Due to fear and misconceptions, healthcare workers even shy away from prescribing insulin because the wrong prescription can cause life-threatening consequences, so they prefer to prescribe oral medications*.

### Lack of T1DM care continuity

Young people, particularly those in urban areas, expressed their dissatisfaction with the frequent turnover of their healthcare professionals, particularly those with whom they have grown to develop cordial relationships. Participants expressed a desire for long-term continuity of care with their healthcare providers. In their assertions, meeting new healthcare providers was a reason for repeated T1DM storytelling and their irregular clinic visits. In explaining this issue, this was what a 21-year-old female warrior with 7 tears of lived experience said:

*Anytime I go to the hospital, I meet new house officers over there and some of them don’t even know* [about T1DM] *such that they must call their bosses* [previous doctors] *to tell them about my situation most of the time. As such, I always must be repeating my story all the time.*

In corroborating this issue, a 24-year-old male warrior with 10 years of lived experience had this to say:

*That Doctor took good care of me until he was transferred. Since then, I haven’t been regular at the hospital because I don’t feel happy going there.*

### Lack of specialist care

All the participant categories were concerned about the lack of trained healthcare providers, particularly in remote and peri-urban areas to manage the complexities of their condition. There was a lack of trained nurses, endocrinologists and diabetologists to provide adequate T1DM care. For instance, a Nurse in charge of a diabetic unit had this to say:

*The hospital lacks most of the specialists involved in the management of diabetes. Currently, there is no trained diabetic nurses or diabetologists in this facility to support the young ones* [Nurse in-Charge of a diabetes unit].

Also, some expert patients who had experienced T1DM services from both a specialist and a general practitioner from different healthcare providers considered specialist care as *“the best”.* In their revelations, this was what a 24-year-old female warrior with 13 years of lived experience had to say:

*I was seen by a general physician which was okay, but ever since I relocated to Accra and started seeing a specialist, I know it’s the best!*

### Shared physical space for T1DM care

During clinical care and hospitalization, participants described being kept in the same room with adults living with type 2 diabetes who had debilitating foot complications and amputations as “so bad”. They showed increased anxiety from being exposed to diabetes complications and recounted stories shared by the adult patients. In explaining this issue, this was what some participants had to say:

*When I go to the hospital, I am mixed with the elderly, those around 40,50 thereabout and they will be saying, eei, this small boy has gotten diabetes, soon they will cut his leg* [a 21-year-old male warrior with 10 years of lived experience].

*I was the only type 1 diabetic and the only kid among them. They were all mothers, fathers and grandparents with wounds which were so bad. I told my parents, and they got a referral for me to go to a different hospital where I saw some young people like me with diabetes* [an 18-year-old female warrior with 17 years of lived experience].

The lack of physical space for T1DM care was an issue of concern for both patients and their caregivers. In corroborating this issue, a 55-year-old mother with 5 years of lived experience had this to say:

*Anytime we went to the hospital, I wasn’t happy because they [patients] were mostly old people.*

### Poor healthcare provider—caregiver interactions

Participants described healthcare providers as generally supportive. They admired healthcare providers' supportive gestures such as facilitating quick access to medications at the healthcare facility, responding to patients' phone calls and text messages, giving them glucometers free of charge, and at times, paying for patients’ transportation back home. Despite this support, some patients and caregivers described instances in which they felt healthcare providers interacted with them poorly, including blaming, name calling and shouting at them. In explaining this issue, this was what a 56-year-old mother with diabetes, and 6 years of lived experience from 2 children living with T1DM had to say:

*We [mother and children with T1DM] went to the hospital, and the doctor looked at me in the eyes and said, madam, why? Madam, why? Take your condition as it is; why have you affected all your children with it? That is, he is accusing me of being a witch who has used diabetes to harm my children.*

### Long waiting time

Long waiting time was a major source of concern as patients and their carers navigated the healthcare system. This theme was identified from sub-themes on hospital bureaucracy, medication queues, and quests for expert consultations. Long waiting time was a major motivator for some young people and their caregivers who could afford to seek private healthcare. In their narrations, participants revealed this:

*Sometimes, there are lots of protocols to go through just to get a vial of insulin* [a 24-year-old female warrior with 19 years of lived experience].

Similarly, a 19-year-old female warrior with 7 years of lived experience shared her recent experience at a public healthcare facility. In her revelations, delay in the public healthcare sector is a reason for her preference for private healthcare facilities. This was what she said:

*The past month I came here, they delayed my time, so I prefer the private clinics because they respond to our needs promptly.*

### Outdated treatment plans

A caregiver shared her concern about the treatment plan her child living with T1DM receives in Ghana. She reported that her child was not receiving contemporary T1DM care. In her narrations, insulin, blood glucose testing and insulin administration measures were outdated.^1^ In explaining this issue, this was what was said:

*Their [healthcare system] treatment plans are outdated. They mostly prescribe Mixtard insulin, which is the most common, and no intermittent short-acting insulin prescription during typical events like partying. These days there are advancements in devices that automatically test glucose and administer insulin which are all lacking here* [a 37-year-old mother with 5 years of lived experience].

### Integration of the barriers to accessing T1DM Care and the chronic care model

The linkages between the themes and the components of the CCM were synthesised and presented in a Matrix Framework. As shown in Table 2, the common healthcare barriers concerned two key components of the CCM which are the delivery system design, and self-management support. Other important areas of concern were the organisation of healthcare, decision support and clinical information systems.

**Table 2:**
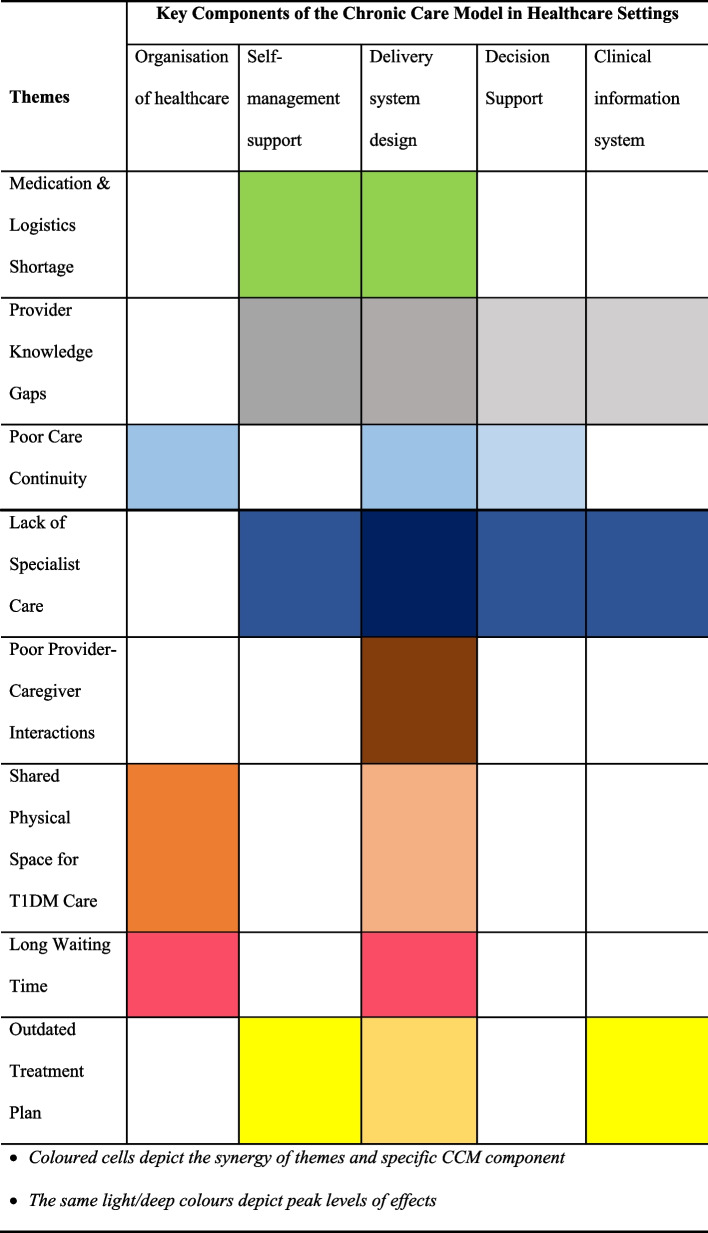
A synthesis of the relationship between identified themes and key components of the CCM

*Coloured cells depict the synergy of themes and specific CCM component*

*The same light/deep colours depict peak levels of effects*

### Proposal for integrated healthcare for T1DM care in low-resource settings

**Fig. 1:**
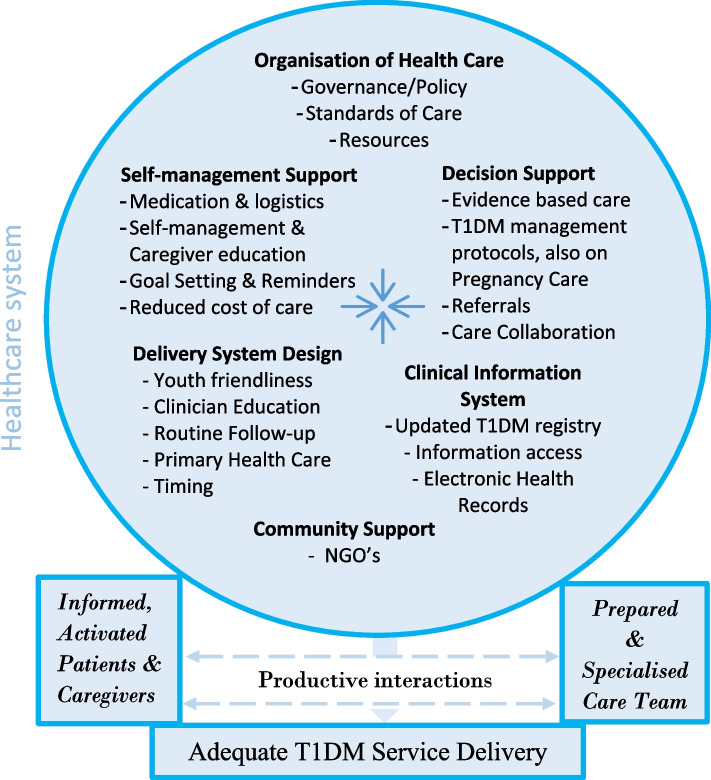
Integrated Healthcare for Type 1 Diabetes Management (IHC-T1DM). Source: Owusu B.A and Doku D.T (2023). Adapted from Wagner et al., [13, 21, 22] and the World Health Organisation [23]

The common health setting barriers faced by young people reflect key components of the CCM. In developed countries where CCM is commonly practised due to improved T1DM resources including diabetes clinics, addressing the barriers in low-resource settings to achieve functional and clinical T1DM management outcomes in healthcare settings will require an integrated model of care. This is because integrated care models tend to be organised, patient-focused, cost-effective, sensitive to change/innovation, and offer benefits to individuals, their families, healthcare teams, the community and health systems in general [29, 30]. Integrated healthcare entails putting together adequate T1DM resources, coordinating care and facilitating easy access to the multidimensional care needs of patients and their caregivers. Acknowledging resource constraints in LMICs, integration will imply identifying diabetes resources including human, material, health insurance, and innovations in healthcare settings and leveraging them to provide improved care for young patients. As shown in Fig. 1 below, we propose an Integrated Healthcare for T1DM (IHC-T1DM) based on the CCM, study results, and fieldwork experiences.

Innovation in healthcare for chronic conditions requires health system attributes such as appropriate healthcare organisation, adequate self-management support, suitable delivery system design, decision support, clinical information systems, and community support, as well as a productive interaction between patients, caregivers’ healthcare providers and the T1DM community. These health systems change components are not distinct entities, rather their limits blur and dynamically influence each other. Innovation is therefore necessary in each change component. The implementation of IHC-T1DM could be enhanced through an evidence-based public health system organisation of T1DM care, and collaboration with private partner organisations through interventions such as the iCARE initiative, and the Diabetes Youth Care through their monthly psycho-social support group meetings. Community support is important to fill in gaps in care (including psychosocial support) to greatly enhance the care experiences for T1DM patients.

## Discussion

Providing healthcare for people living with T1DM requires plans that make clinical, practical, and emotional sense to patients. In this sense, T1DM care entails clinical endeavours by healthcare providers to identify healthcare barriers faced by patients, and to determine how best to respond to them [31]. To this end, we explored the health-setting barriers to adequate T1DM care and adapted the CCM to propose a model of T1DM care in healthcare settings. The results show that young people living with T1DM and their caregivers face multiple barriers in healthcare facilities. The commonest barriers were shortage of life-saving insulin and T1DM management logistics, healthcare provider knowledge gaps, lack of care continuity, and lack of specialists’ care. Other barriers were poor healthcare provider-caregiver interactions, shared physical space with adult T2DM patients, long waiting times, and outdated treatment plans.

These findings are consistent with earlier research across different countries and population sub-groups [9–11, 28–33], emphasizing common barriers of T1DM care, yet provide novel evidence, demonstrating the marked challenges faced by young T1DM patients in Ghana. Consistent with our results, young people living with T1DM faced barriers such as insulin shortages, inadequate professional care, and long waiting times [32–36]. For young people living with T1DM to achieve ideal glycaemic control and stay alive, access to life-saving insulin is critical. In like manner, T1DM management supplies including glucometers, test strips, and adequate professional care cannot be overemphasised. The barriers reported in this study can adversely affect access and use of healthcare services, as well as expose patients to health-compromising behaviours. For instance, in a related study, Owusu et al. found that the lack and/or erratic supply of life-saving insulin, and repeated use of syringes were major reasons for insulin rationing and abscesses among young people living with T1DM respectively [9].

As confirmed in other studies, the lack of access to professional care is a major reason for the failure of young people living with T1DM to utilise healthcare facilities [9–11, 37]. Young people living with T1DM who lack access to professional care have increased length of hospital admissions, worsened glycaemic control, and poor health outcomes [38]. Similarly, limited knowledge of some healthcare providers about adequate T1DM management, especially during major life events such as managing T1DM during pregnancy has been confirmed in other studies [5, 9, 39]. We found that some healthcare providers' inability to detect or adequately treat T1DM during pregnancy resulted in foetal damages which have long-term adverse health and socio-economic impact on young mothers. Knowledge gaps among healthcare providers have also been identified elsewhere [40–42]. In China, a similar immense gap was identified between the needs of pregnant women with T1DM and current care provision, necessitating their call for professional and multidisciplinary support to optimise pregnancy care for women with T1DM [43]. Diabetes management education by certified diabetes healthcare providers has been found to improve patients’ self-management knowledge, psychosocial, and clinical outcomes [19, 44]. Similarly, an adequate healthcare delivery system allows for better communication, which is a known behavioural therapy to reduce glycosylated haemoglobin (HbA1C) levels. It ensures proper adjustment process and provides stronger support networks for persons living with diabetes [45, 46].

Young people living with T1DM prefer to have continuity of care with their healthcare providers [47, 48]. Continuity of care with healthcare providers can serve as a major pathway to improve healthcare utilisation, and consequently improve glycaemic control. For instance, care continuity with healthcare providers help to identify persons at risk of complications early in their disease management [49, 50]. Further, poor healthcare organisation adversely affects HbA1C levels, and disrupts adequate eye and foot care [46, 51].

Other barriers such as poor interaction between healthcare providers and caregivers, exposure to debilitating complications due to poor T1DM/age-appropriate/condition-specific space, long waiting time and outdated treatment plans shipwrecks acceptable practices for adequate T1DM management. Considering the age and developmental needs of young people living with T1DM, keeping them in the same physical space as older people facing debilitating complications can be traumatising. Generally, the few resources available for T1DM care are compromised by the lack of trained staff, limited space for age-appropriate admissions, and outdated treatment plans. Although the blame and shame attitude of some healthcare providers towards parents/guardians of T1DM patients is a misnomer, such evidence has been found in some remote regions in British Columbia [52].

As noted, addressing these healthcare barriers in LMICs will require the adoption of integrated healthcare. Integrated healthcare can be beneficial for people with diabetes by providing safer spaces to promote adequate health-seeking behaviour in a more organised and sustainable manner. Through primary healthcare mechanisms in Ghana, patients and their caregivers could benefit from integrated mechanisms that bridge the gap between access and use of healthcare for diabetes care. For instance, the integrated healthcare approach has been developed in Sweden to meet the needs of patients by linking primary care, hospital care and community care via a chain of pathways based on agreement between care providers [53]. The IHC-T1DM model is therefore necessary and can contribute to the goal of reducing by a third premature death from NCD’s including diabetes by 30% (Sustainable Development Goal (SDG) 3.4, achieve universal health coverage, including access to quality essential healthcare services (SDG 3.8), and reduce inequalities for all (SDG 10) as contained in the United Nations SDGs [54].

### Strengths and limitations of the study

A major strength of this study lies in its methodology, theoretical and conceptual contributions. The study employed interpretivist philosophies to uncover the barriers faced by young patients at healthcare facilities. Data sources and methods were triangulated across four categories of participants. By employing blended methods, robust and cross-cutting evidence was analysed to understand the subject matter. Theoretically, this study proposes and makes a meaningful contribution to health services research through the Integrated Healthcare for T1DM (IHC-T1DM). A major limitation of this study is that the IHC-T1DM will benefit from knowledge co-creation between healthcare providers, patients, and caregivers about the best ways to deliver adequate T1DM care to patients.

### Implications for policy, practice and future research

Care needs for people living with chronic conditions differ from the needs of those important for acute conditions. Chronic conditions require more than biomedical care to include care that cuts across space and time, multi-disciplinary team of care providers, and systems that are sensitive to cost. The results call for the need to train healthcare providers to support T1DM care and ensure easy access to insulin and T1DM management logistics. As a developing country undergoing its epidemiological transition, T1DM, compared to T2DM affects a relatively smaller number of people in Ghana. Despite T1DM's disruptive effects on patients, they show optimism in improving and participating in interventions to improve their health [55]. With the ongoing introduction of improved technology in some major healthcare facilities in Ghana, the use of electronic health record systems for example will enhance continuity of care through timely access to patient’s medical records, identify predictable complications early, and encourage a productive interaction between the healthcare provision team. Without innovations, health systems will grow ineffective as chronic conditions continue to rise. Innovation is important to ensure that the billions of diabetes expenditure leads to an actual improvement in health outcomes and patients’ quality of life.

Due to the peculiar needs of young patients, care integration must ensure the provision of age-appropriate services/resources in a youth-friendly manner. Acknowledging resource constraints in LMICs will imply providing youth clinics that are linked to professional service providers. Also, successful healthcare and community programmes can be leveraged upon and /or integrated to improve care for T1DM patients. Similarly, a planned and gradual approach to a sustainable IHC-T1DM implementation is advised. In the case of chronic conditions, patients’ needs are similar. Thus, the IHC-TIDM can be adapted to improve care for other chronic conditions in low-resource settings. There is a need for future studies to co-create knowledge between healthcare providers, patients, and caregivers about the best ways to deliver T1DM care provision to patients.

## Conclusion

Young people living with T1DM, and their caregivers face multiple barriers of care in healthcare settings. These barriers were mostly due to the healthcare system delivery design, inadequate self-management support, poor organisation of T1DM care including poor coordination, limited clinical information systems, and inadequate decision support which are key components of the CCM. There is a need to address these barriers to T1DM care, and this could be optimised by implementing an integrated approach to T1DM care by organising and leveraging available health system resources.

### Supplementary Information

**Additional file 1. **Interview Guide.

## Data Availability

The datasets used and/or analysed during the current study is available from the corresponding author on reasonable request.

## Footnotes

1 This mother previously sought healthcare for her son living with T1D from the United States; from where they migrated to stay in Ghana. As such, she is much exposed to improved means of caring for young people living with T1D.
