# Supplementary material for: Towards an integrated type 1 diabetes management in low-resource settings: barriers faced by patients and their caregivers in healthcare facilities in Ghana
Source: BMC Health Serv Res. 2024 Jan 4;24:21. doi: 10.1186/s12913-023-10410-0 (PMC10768474; doi:10.1186/s12913-023-10410-0)
Supplement: Supplementary file 1 — Additional file 1. Interview Guide. [file 12913_2023_10410_MOESM1_ESM.pdf]

LIVED EXPERIENCES OF YOUNG PERSONS WITH TYPE 1 DIABETES AND THEIR CAREGIVERS IN SOUTHERN GHANA

IN-DEPTH INTERVIEW GUIDE FOR YOUNG PERSON'S

Introduction: Introduce yourself and seek for their informed consent prior to the start of Interview.

**SECTION A: Socio-demographic characteristics of participants**<br>Could you please tell me about yourself? Ask about	
1. *Age*	2. *Sex*
3. *Duration of diabetes*	4. *Religious affiliation*
5. *Place of residence*	6. *Marital status*
7. *Level of education*	8. *Occupation*
9. *Primary caregiver*	10. *Family history of diabetes?*
11. *Caregiver's Occupation*	12. *Insulin injection per day*
13. *Telephone number*	14. *Interview start date*
15. *Interview end date*	16. *Duration of interviews*
17. *Interviewer*	18. *Etc.*

SECTION B: Lived Experiences of young persons with type 1 diabetes

PREAMBLE: *This section seeks to solicit for information about* ***your*** *lived experiences with T1D. It seek information about your diagnosis, experiences and T1D journey since diagnosis.*

1. How did you get to know that you have diabetes?
   a. *If not mentioned, what were the signs and symptoms before you found out you have diabetes?*
   b. *Where (home, hospital etc.) were you diagnosed of diabetes and who diagnosed you?*
   c. *How timely was your diagnosis, any admission?*
   d. How did you feel when you were diagnosed of diabetes and ***why***?

2. How has your family/primary caregiver related to you since your diagnosis? [*Ask for all forms of support, intermittent or continuous?*
   a. *What are your family beliefs and approaches to manage your condition?*
   b. *What has been the supportive role/challenge of any girlfriend/boyfriend if any*

3. What has been your experience with the healthcare system? [*Ask for place of healthcare, accessibility, frequency of visits, attitude of practitioners, reason for visits, access to essential drugs, acceptance of NHIS, referrals etc.*]
   a. How will you describe your satisfaction with the services you receive at the healthcare center? [Ask: are they tailored to your specific needs)
   b. Any stories to share?

4. How have your school/workplace related with you since your diagnosis? *Probe about safe spaces, forms of support whether intermittent or continuous]*

SECTION C: Knowledge about Type 1 Diabetes Management

**<u>PREAMBLE:</u>** *Effective diabetes management requires access and use of specific T1D knowledge. This section seeks to solicit for your information on diabetes knowledge, sources of information, and how your knowledge levels can be improved.*

5. How knowledgeable are you when it comes to the management of your condition? [*Probe for blood glucose monitoring, exercise, diet, causes of T1D etc.*]
    a. In a typical day when you have low blood glucose/sugar, describe what you do to respond to and manage this situation? [for every practice raised, ask why]
    b. How do you respond to and manage high blood sugar level? [for every practice raised, ask why]
    c. How do you manage diabetes during sick days? [for every practice raised, ask why]

6. Where do you learn about the management of your condition [mention all mediums you use to learn about your condition] <u>Ask about why the sources each?</u>
    a. Can you tell me about the things you learn about?

7. Which area(s) of diabetes management do you need more knowledge and why?
    a. What do you want to know about responds to Q7

8. What can be done to improve your knowledge on effective diabetes management?

9. What has been the role of:
    a. Family members, friends etc. in supporting you to manage your condition
    b. Support groups in supporting you to manage your condition

**SECTION E: Challenges and Coping strategies**
**PREAMBLE:** *Young persons living with T1D face challenges with their diabetes management including access to healthcare. To cope with such problems, young people adopt different coping strategies to lessen the impact of T1D on their general health and wellbeing which differ from person to person. This section seeks to solicit for information about the challenges and young people living with T1D face in managing their T1D, as well as their coping strategies. Coping strategy simply means what you do when faced with such challenges.*

10. What are the challenges you face as a result of your diabetes? For each mentioned, ask about the coping strategy. Eg.

**a. Challenges**	**b. Coping Strategies**
If not mentioned, ask about each of the following challenges, and coping strategies	
➢ Difficulty in blood glucose monitoring and injection: <u>*Why is this a challenge?*</u>	How do you cope with this challenge? *Any story to share?*
➢ Access to diabetes management information: <u>*Why is this a challenge?*</u>	How do you cope with this challenge? *Any story to share?*
➢ Access to healthcare: <u>*Why is this a challenge?*</u>	How do you cope with this challenge? *Any story to share?*
➢ Access and Storage of life-saving insulin: <u>*Why is this a challenge?*</u>	How do you cope with this challenge? *Any story to share?*
➢ Coverage of NHIS: <u>*Why is this a challenge?*</u>	How do you cope with this challenge? *Any story to share?*

➢ Stigma and discrimination: *Why is this a challenge?*	How do you cope with this challenge? *Any story to share?*
➢ Adequate nutrition: *Why is this a challenge*	How do you cope with this challenge? *Any story to share?*
➢ *Covid-19 challenges: Why is this a challenge?*	How do you cope with this challenge? *Any story to share?*

11. How can the lived experiences of young persons be improved?

Closing courtesies

Thank you for your time and information. Can I please come back or call you for additional information if there is the need?

NB: Sections which were not directly related to this paper are omitted.

IN-DEPTH INTERVIEW GUIDE FOR PARENTS/CAREGIVERS

SECTION A: Socio-demographic characteristics of participants

A. Can you tell me about yourself: *[Age, sex, occupation, place of residence, family history of diabetes, income, educational level, marital status, parity, years involved in T1D care?]*

SECTION B: Parents Experiences with T1D

PREAMBLE: Raising a child/ward living with T1D presents an additional task to parents. This section seeks to solicit for information about your experiences with raising a diabetic child.

12. Can you tell me about your child/ward who has diabetes? [*Age, sex, date of diagnosis, signs and symptoms prior to diagnosis, where it was diagnosed, who diagnosed it etc.*]
13. How did you feel when you were told your child/ward has diabetes? [*Probe for knowledge about diabetes causes, beliefs, worries, etc.*]
14. What has been your memorable experiences over the past years? [*ask about: both the good and bad experiences*]
    a. How does it feel to be participating in your child/wards T1D management process of diabetes as a parent? [*Task of work, conflicts, mental images, stress, etc.*]

SECTION C: Knowledge Provision

PREAMBLE: *Parental/Guardian knowledge can enrich the knowledge of their wards in managing diabetes. Parental knowledge can also influence T1D management approaches and outcomes. This section seeks to solicit for information about your knowledge on diabetes and how such knowledge can be improved.*

15. What are the sources of your T1D information? [ask: formalized or through experiential learning?
    a. Can you tell me about the things you learn about and why? Are they tailored to the specific needs of your child?
    b. How well are you informed with the knowledge to adequately manage T1D?
16. What can be done to improve your knowledge in order to help you to manage your child's condition? [Ask for homecare visits by educators, role-plays during meetings etc.]

SECTION E: Challenges faced by parents/caregivers and coping strategies

PREAMBLE: Raising a child/ward living with diabetes can be challenging, and parents adopt several situations in the process of T1D management. This section seeks to solicit information about the challenges you face as a parent and your adaptation strategies.

17. What are the challenges you face as a parent in providing diabetes care? [*Probe for access to information, financial burden, increased stress, distortion of plans etc.*]
    a. How do you adapt/cope?
    b. How can each of these challenges be addressed?
18. What are the challenges your child face as a result of his condition? [*Probe for educational, informational, relational as well as clinical outcomes etc.*]
    a. How do you adapt/cope?
    b. How can each of these challenges be addressed?
19. What are the community/church/friends-related challenges you have encountered? [*Ask about community support, stigma and discrimination etc.]*
    a. How do you cope?

    b. How can each of these challenges be addressed?

20. What are the health system challenges you have encountered? [*Ask about challenges with: diagnostic technologies, human resources, insulin provision, diabetes technologies, primary health care, NHIS service coverage etc.*]
    a. How do you cope?
    b. How can each of these challenges be addressed?

Closing courtesies

Thank you for your time and information. Can I please come back for additional information if there is the need? Once again, thank you.

NB: Sections which were not directly related to this paper are omitted.

IN-DEPTH INTERVIEW GUIDE FOR DIABETES NURSE/PROVIDER

SECTION A: Basic socio-demographic characteristics of participant

Can you please tell me about yourself *[Ask: Position, name of hospital, duration of working with T1D patients, in what capacity*

SECTION B: Health System Context

21. Can you tell me about your role as a diabetes nurse? [*Probe for information support, psychosocial support etc*.]
    e. What has been the trend of T1D diagnosis in the facility [age, sex, family structures etc.]
    f. Can you tell me about the hospitals human resource capacity for managing diabetes among young people? [*Probe for number of trained nurses, diabetelogists, nutritionists, physicians, endocrinologist in the unit?*]
    g. What are the services and resources in this facility for managing diabetes among young people? [*Probe for diabetes unit, registers, diagnostic tools, access to insulin, coverage of NHIS, diabetes technologies, referrals etc.]*
    h. What complications do young people present to this facility? Why do they come about?

SECTION C: Knowledge Provision

22. What specific information-related services are given to adolescents and young persons living with T1D? [*Probe for blood glucose monitoring, exercise, diet, insulin injection etc.*]
    a. How are these information services conveyed? [Probe for mediums, time, frequency, structured or unstructured, individualized or group information etc.]
    b. Does the information provided has evaluation mechanisms as part of the process? If No, ask: How do you assess knowledge comprehension and application of the information by AYP's?
23. What is the structure for continuous learning and information provision? [Probe for sources of information, formalized or through personal knowledge?
    a. Can you tell me about the things you learn about? Are they tailored to the specific needs of young people?
24. How do young people learn about their insulin and its technologies? [probe for glucometers, syringes, keeping insulin, insulin injection etc.]
25. What can be done to improve the knowledge levels of AYPs to manage T1D? [Ask for diabetes applications, homecare visits by educators, role-plays during meetings etc.]
26. How is the relationship between healthcare providers and AYP's? [Ask whether they are too protective, worry about their diabetes etc]
    a. How do such relationships affect the HRQoL of AYP's? [Any story to tell?]

SECTION E: Challenges faced by health systems in managing T1D

27. What are the personal challenges you face as a healthcare provider in providing diabetes care? [*Probe for access to information, dealing with parents and young people, any financial burden etc.*]
28. What are the health system challenges faced in providing diabetes care? [*Ask about challenges with: diagnostic technologies, human resources, insulin provision, diabetes technologies, primary health care, NHIS service coverage, funding etc.*]

29. How can these challenges be addressed?
    a. probe for addressing healthcare-related challenges such as information, insulin, access, PHC, NHIS
    b. probe for addressing social-related challenges such as financial cost, stigma & discrimination, family worries etc.]

SECTION F: Coping styles/Strategies

30. In all these personal challenges, how do you cope? [Refer to challenges and ask each]
31. With all these health system challenges, how do healthcare providers cope? [Refer to challenges and ask each]
32. What has been the development in this facility in providing diabetes care to AYP's?

ASK*:* *Is there any information you may want to add as I bring this interview to an end?*

Closing courtesies

Thank you for your time and information. Can I please come back for additional information if there is the need? Once again, thank you.

NB: Sections which were not directly related to this paper are omitted.
